# Lifelong Football Training: Effects on Autophagy and Healthy Longevity Promotion

**DOI:** 10.3389/fphys.2019.00132

**Published:** 2019-02-19

**Authors:** Annamaria Mancini, Daniela Vitucci, Morten Bredsgaard Randers, Jakob Friis Schmidt, Marie Hagman, Thomas Rostgaard Andersen, Esther Imperlini, Annalisa Mandola, Stefania Orrù, Peter Krustrup, Pasqualina Buono

**Affiliations:** ^1^Dipartimento di Scienze Motorie e del Benessere, Università degli Studi di Napoli Parthenope, Naples, Italy; ^2^CEINGE-Biotecnologie Avanzate, Naples, Italy; ^3^IRCCS SDN, Naples, Italy; ^4^Department of Sports and Clinical Biomechanics, Sport and Health Sciences Cluster, University of Southern Denmark, Odense, Denmark; ^5^Copenhagen Centre for Team Sport and Health, Department of Nutrition, Exercise and Sports, University of Copenhagen, Copenhagen, Denmark; ^6^Health Sciences, College of Life and Environmental Sciences, St. Luke’s Campus, University of Exeter, Exeter, United Kingdom

**Keywords:** football, lifelong training, longevity, cardiovascular capacity, autophagy

## Abstract

Aging is a physiological process characterized by a progressive decline of biological functions and an increase in destructive processes in cells and organs. Physical activity and exercise positively affects the expression of skeletal muscle markers involved in longevity pathways. Recently, a new mechanism, autophagy, was introduced to the adaptations induced by acute and chronic exercise as responsible of positive metabolic modification and health-longevity promotion. However, the molecular mechanisms regulating autophagy in response to physical activity and exercise are sparsely described. We investigated the long-term adaptations resulting from lifelong recreational football training on the expression of skeletal muscle markers involved in autophagy signaling. We demonstrated that lifelong football training increased the expression of messengers: RAD23A, HSPB6, RAB1B, TRAP1, SIRT2, and HSBPB1, involved in the auto-lysosomal and proteasome-mediated protein degradation machinery; of RPL1, RPL4, RPL36, MRLP37, involved in cellular growth and differentiation processes; of the Bcl-2, HSP70, HSP90, PSMD13, and of the ATG5-ATG12 protein complex, involved in proteasome promotion and autophagy processes in muscle samples from lifelong trained subjects compared to age-matched untrained controls. In conclusion, our results indicated that lifelong football training positively influence exercise-induced autophagy processes and protein quality control in skeletal muscle, thus promoting healthy aging.

## Introduction

Aging is a physiological process characterized by a progressive decline of biological functions and a progressive increase of destructive processes in cells and organs, eventually leading to death (López-Otín et al., 2013; Aunan et al., 2016; Flatt and Partridge, 2018).

This process not only affects the homeostasis within organs and the tissue response to injury, but, at a molecular level, it is associated with accumulation of DNA and protein damage (Soares et al., 2014; Boros and Freemont, 2017). Several physiological changes occur with aging, the most notable are: the decrease of cardiac output at rest and maximum breathing capacity, decrease in renal filtration rate and in nerve conduction velocity (McClaran et al., 1995; Glassock and Winearls, 2009; Strait and Lakatta, 2012; Jafari Nasabian et al., 2017). Additionally, aging acts also on the metabolically active bone and muscle tissues by triggering events that eventually impair their cross-talk determining low body mass density (BMD) and/or sarcopenia (Boirie, 2009). At this regard, fatty infiltration in both tissues is considered an age-related hallmark leading to osteosarcopenia, a recently defined geriatric syndrome, which is associated to increased morbidity and mortality risk among older people (Hirschfeld et al., 2017). Among the shared patho/physiological pathways, bone and muscle tissues can both adapt to mechanical stimuli, these latters being able to regulate the delicate balance between osteogenesis and osteolysis and between muscle growth and breakdown (Isaacson and Brotto, 2014; Kawao and Kaji, 2015). Nowadays, it is well recognized the beneficial impact of physical activity on multiple organ functions, by providing an adeguate and efficient mechanical stimulus on all the components of the locomotor apparatus (Radak et al., 2018). Physical Activity (PA) and exercise training delays functional decline in most of the biological pathways in older people, and plays a key role in promoting healthy longevity (Booth et al., 2012; Pedersen and Saltin, 2015). PA and exercise has been shown to affect the skeletal muscle expression of markers involved in longevity pathways differently depending on the exercise mode. As such, it has been shown that long-term aerobic training increased the expression/activity of SIRT1 and SIRT3 in older rats (Ferrara et al., 2008; Palacios et al., 2009), whereas resistance training increased the expression of Heat Shock Proteins (HSPs) in rat skeletal muscle (Murlasits et al., 2006). Furthermore, the expression of HSPs in skeletal muscle following exercise induced stress appears to be intensity dependent, with higher exercise intensities producing a greater physiological response (Gjøvaag and Dahl, 2006; Folkesson et al., 2008; Paulsen et al., 2009, 2012). Also, HSPs induction is requested for the maintenance of cellular homeostasis and promotion of longevity (Lindquist and Craig, 1988; Hartl, 2016).

Recently, a new mechanism, autophagy, was indicated in the adaptation induced by acute and chronic exercise as being responsible for positive metabolic adaptation and longevity promotion (Vainshtein and Hood, 2016). Autophagy is an ubiquitous catabolic process which leads to the degradation of cytoplasmic components in the cells. This process plays a crucial role in the physiological turnover of most proteins, biological membranes, mitochondria, and ribosomes (Park and Cuervo, 2013). Autophagy impairment may ultimately lead to the accumulation of damaged cellular components, including mitochondria and protein aggregates and is associated to the aging process both in invertebrates and higher organisms (Cuervo et al., 2005; Terman and Brunk, 2006; Schiavi and Ventura, 2014; Sakuma et al., 2017).

The acute physiological response to a recreational football training session in elderly subjects is characterized by a high aerobic load, with mean and peak heart rate (HR) reaching 84–88% and 93–98% of individual maximum heart rate (HRmax), respectively (Randers et al., 2010; Andersen et al., 2014). Also, during a recreational football training session in elderly subjects, significantly elevated blood lactate levels have been reported (Andersen et al., 2014). This finding supports that lactate production is high during a football training session, and that the intense and frequently changing activity pattern observed during a football training session activates glycolysis to a greater extent than continuous walking and straightforward running performed at similar or higher average movement speed (Drust et al., 2000). The above findings are comparable to what can be observed in elite football players (Krustrup et al., 2006).

Long-term recreational football training increases skeletal muscle fat oxidation and anti-oxidative potential, stimulates musculoskeletal metabolic adaptations, and results in beneficial improvements in the cardiovascular system (Krustrup et al., 2010; Alfieri et al., 2015; Bangsbo et al., 2015; Schmidt et al., 2015; Andersen et al., 2016; Krustrup and Krustrup, 2018). However, up to now, the molecular effects of exercise training on regulation of autophagy and processes involved in longevity promotion are not completely elucidated, and no consensus is established about the different gene/protein expression in response to exercise (Koltai et al., 2012; Kim et al., 2013; Konopka et al., 2014; Vainshtein et al., 2014; Moreira et al., 2017).

Very recently, increased expression of key markers involved in mitochondrial biogenesis, oxidative metabolism, and DNA-repair/senescence suppression pathways were found in skeletal muscle from Veteran Football Players (VPG) compared to untrained elderly subjects (Mancini et al., 2017). As such, the aim of the present research was to investigate, through a differential transcriptomic approach, the effects of lifelong recreational football training on the expression of key markers involved in the autophagy response for the maintenance of protein quality control, related to healthy longevity promotion, in skeletal muscle from VPG compared to elderly untrained control subjects.

## Materials and Methods

### Subjects

Thirty healthy males aged 65–77 years volunteered to take part of the study. The subjects were divided into two groups according to their previous experience as football players, with one group consisting of veteran football players (VPG; *N* = 15), and one group consisting of healthy age-matched untrained controls (CG; *N* = 15) (anthropometric and clinical characteristics are reported in Table 1). VPG was recruited via direct contact to local football clubs in the greater Copenhagen area, and had on average been active as football players for 52 ± 11 years (median 58 years, range 25–62 years) and had been training one session per week (1.5 ± 0.6 h/session) and played 26 ± 12 football matches (2 × 35 min) per year for the last 10 years as previously reported (Schmidt et al., 2015). CG was recruited via advertisement in local newspapers, and none of the subjects had been involved in regular physical exercise training during a major part of their adult life. In addition, the participants reported that they had been primarily inactive for the past 5–10 years.

**Table 1:** Anthropometric and clinical characteristics of subjects participating to the study.

	VPG	CG
Number of subjects	15	15
Age (yrs)	69.3 ± 3.2	68.3 ± 2.8
Height (cm)	178.5 ± 4.9	176.8 ± 6.3
Body weight (kg)	77.4 ± 7.7	85.9 ± 11.2*
BMI (kg/m^2^)	24.3 ± 2.2	27.5 ± 3.7*
Total body fat (%)	22.9 ± 6.7	30.8 ± 4.6*
VO_2_peak (mL/kg/min)	33.7 ± 5.4	27.6 ± 4.9*

^∗^*p < 0.05 VPG *vs* CG. Values are reported as means±SDs*.

All subjects were informed verbally and in writing about any potential discomforts or risks related to the experimental study protocol, and signed an informed written consent prior to their enrolment in the study. For both groups, inclusion criteria were eligibility to all testing procedures (see below). Exclusion criteria were history or symptoms of cardiovascular disease or cancer, type 2 diabetes, hypertension, nephropathy, or musculoskeletal complaints that were considered to preclude testing. All medical screening procedures were performed by a medical doctor. The study was conducted according to the Declaration of Helsinki and was approved by the local ethical committee of Copenhagen; H-1–2011-013. ClinicalTrials.gov identifier: NCT01530035.

### Habitual Fitness Level

To estimate the daily physical activity level of all participants in CG and in VPG besides the football training sessions and matches, participants filled a questionnaire to quantify all sporting activities and everyday activities. Physical activity was defined as hours spent walking, jogging, running, swimming, biking, golfing, or gardening in order to relate to current standards of advised physical activity in elderly citizens. In VPG, nine subjects did not participate in other sporting activities besides football, whereas the remainder reported activities for 2.0 ± 1.5 h/week besides football training consisting of golf, running, biking, fitness, or kayaking. Additional physical activities or no additional physical activities besides football training in VPG were not associated with higher VO_2_peak values as previously reported (Schmidt et al., 2015). In CG, the subjects were on average active 2.5 ± 3.1 h/week (median 2 h, range 0–10 h/week) mainly consisting of everyday activities such as housing and gardening, gymnastics, walking, golf, swimming, and no activities. Thus, the majority of the physical activities in CG did not amount to a formal moderate-intensity aerobic level^1^. Two subjects in CG reported more than 5 h of physical activity but were active with walking and golf, i.e., low-intensity activities. As such, in CG, a large variance in habitual physical activity levels was observed. However, habitual physical activity in CG was not significantly associated with higher cardiorespiratory fitness (Schmidt et al., 2015).

### Muscle Sample Collection

Muscle biopsies were obtained under standardized conditions after an overnight fast between 7 and 10 a.m. from the vastus lateralis under local anesthesia (1% Lidocaine, Amgros 742122, Copenhagen, Denmark) using the Bergstrom technique. The muscle sample (40 mg wet weight) was immediately frozen in liquid N_2_ and stored at -80°C until further analysis. All subjects were instructed to refrain from strenuous physical activity or exercise training 48–72 h prior to the invasive procedure in addition to consuming a standardized carbohydrate-rich meal the night before reporting to the laboratory as previously described (Andersen et al., 2016).

### Body Composition

Whole body fat percentage was determined by whole body Dual energy X-ray absorptiometry (DXA) scanning (Prodigy Advance, Lunar Corporation, Madison, WI, United States). Scanning was performed between 7 and 10 a.m. under standardized conditions after an overnight fast. All DXA scans were performed by the same experienced observer and the DXA software regional cut-points were visually inspected and manually adjusted if necessary. Body height and body weight were measured on standard scales with subjects wearing light clothes and BMI (kg/m^2^) was subsequently calculated.

### Peak Oxygen Uptake

For determination of peak oxygen uptake (VO_2_peak) an incremental cycling test to exhaustion was applied. During the incremental cycling test, subjects started exercising at a work pace and load of 80 rpm and 40 W, respectively, after which the work load was increased by 20 W every 2-min until volitional fatigue. Pulmonary gas exchange (OxyconPro; VIASYS Healthcare, Höechberg, Germany) was measured continuously throughout the exercise protocol. VO_2_peak was determined as the highest value achieved during a 30-s period.

### Transcriptome Assay in Muscle Biopsies Samples

RNA extraction has been performed as previously described (Mancini et al., 2017). Total RNA was extracted from the muscle biopsies using an miRNeasy^®^ kit (Qiagen, Hilden, Germany) according to the manufacturer’s instructions. The RNA integrity number (RIN) of samples was assessed using a Bio-Rad Experion automated electrophoresis station (Hercules, CA, United States) before cDNA synthesis. All RNA samples passed the criterion of RIN ≥7. For strong, unbiased results, pooled RNA libraries were produced by evenly pooling six RNA samples, which resulted in three pooled CGs (named CG1, CG2, and CG3) and three pooled VPG libraries (named VPG1, VPG2, and VPG3). The pooled RNA samples underwent microarray assay to determine gene expression profile. In this study, we carried out profiling with GeneChip^®^ Human Transcriptome Array 2.0 (HTA 2.0, Affymetrix, Santa Clara, CA, United States). The RNA samples were prepared using the WT PLUS Reagent kit, followed by hybridization on HTA 2.0 microarray chips. 100 ng of total RNA were subjected to two cycles of cDNA synthesis with the Affymetrix WT PLUS expression Kit. The first cycle (first strand synthesis) is performed using an engineered set of random primers that exclude rRNA-matching sequences and include the T7 promoter sequences. After second-strand synthesis, the resulting cDNA is *in vitro* transcribed with the T7 RNA polymerase to generate a cRNA. This cRNA is subjected to a second cycle – first strand synthesis in the presence of dUTP in a fixed ratio relative to dTTP. Single strand cDNA is then purified and fragmented with a mixture of uracil DNA glycosylase and apurinic/apyrimidinic endonuclease 1 (Affymetrix) in conjunction with incorporated dUTPs. DNA fragments are then terminally labeled by terminal deoxynucleotidyl transferase (Affymetrix) with biotin.

The biotinylated DNA was hybridized to the Human Genechip HTA 2.0 Arrays (Affymetrix), containing more than 285.000 full length transcripts covering 44.700 coding genes and 22.800 non-coding genes selected from H. sapiens genome databases RefSeq, ENSEMBL, and GenBank. Chips were washed and scanned on the Affymetrix Complete GeneChip^®^ Instrument System, generating digitized image data (DAT) files. The data discussed in this publication have been deposited in NCBI’s Gene Expression Omnibus (Edgar et al., 2002) and are accessible through GEO Series accession number GSE125830^2^.

### Bioinformatic Analysis

Genomic data were subjected to Database for Annotation, Visualization and Integrated Discovery (DAVID)^3^ and Ingenuity Pathways Analysis (IPA) (Ingenuity System^4^) to identify and explore relevant biological networks. Genes were uploaded as a tab-delimited excel file of Gene Symbol and Fold Change and mapped to corresponding gene objects stored in the IPA.

### RNA Extraction and RT*q*PCR

Total RNA was extracted from the muscle biopsies and integrity assessed as described above. For reverse transcription-PCR (RT-PCR) analysis, 0.5 μg of total RNA was used in the reaction with SuperScript reverse transcriptase (Life Technologies). The resulting cDNA was analyzed by realtime quantitative PCR (RTqPCR) performed with the iQ5- iCycler Optical System (Bio-Rad, Hercules, CA, United States). IQ SYBR Bio-Rad protocol (100 mM KCl, 40 mM Tris–HCl pH 8.4, 0.4 mM dNTPs, iTaq DNA polymerase 50 U/ml, 6 mM MgCl2, SYBR Green I, 20 nM fluorescein, stabilizer) was applied according to the manufacturer’s instructions. Reaction mixtures were incubated at 95°C for 30 s, followed by two cycles at 95°C for 30 s and 95°C for 3 min and by 40 cycles at 95°C for 15 s and 60°C for 1 min. Finally, 80 cycles were run starting at 55°C and increasing the temperature by 5°C every 10 s up to 95°C. Fluorescence signals were measured during the elongation step. All measurements were performed in duplicate. The target mRNA expression levels were normalized to the levels of the polymerase (RNA) II (DNA directed) polypeptide A (Pol2A) gene using the 2^-ΔΔCT^ method as described in (Vitucci et al., 2018). The Oligonucleotide primer sequences used in RTqPCR are reported below:

      
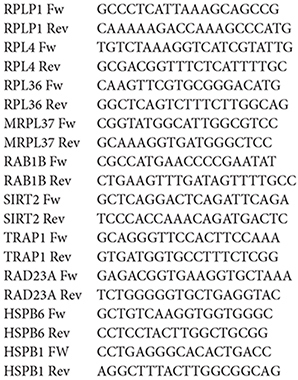


### Western Blotting

The muscle biopsies were mechanically pulverized and protein extraction was performed as previously described (Mancini et al., 2017). Briefly, protein samples (50 μg each) were separated on 4–20% precast gradient polyacrylamide gels (Bio-Rad), transferred to the Hybond ECL nitrocellulose membrane (GE Healthcare) and checked by Ponceau S staining to verify equal loading. The membranes were immunoblotted using rabbit polyclonal antibodies against autophagy related 5 homolog (ATG5), autophagy related 12 homolog (ATG12), mouse monoclonal antibodies against Heat Shock Protein 90 (HSP90), Heat Shock Protein 70 (HSP70), B-cell lymphoma 2 (Bcl-2) (Elabscience, 1:500), monoclonal rabbit antibody proteasome 26S subunit non-ATPase 13 (PSMD13) (Abcam, 1:1000), monoclonal mouse antibody against glyceraldehyde- 3-phosphate dehydrogenase (GAPDH) (1:1000; Santa-Cruz Biotechnology Inc.). Blots were incubated with appropriate horseradish peroxidase-conjugated secondary antibody and target proteins were visualized by ECL detection (GE Healthcare). Densitometric measurements were carried out using Quantity One software (Bio-Rad) as reported elsewhere (Imperlini et al., 2015). GAPDH protein was used to estimate the total amount of loaded proteins. Results were normalized as a percentage of the mean of controls in each membrane.

### Statistical Analysis

Group comparisons were investigated with the application of a one-way ANOVA statistical model.

Microarray data analysis: DAT files were analyzed by Expression Console (Affymetrix Inc.). The full data set was normalized by using the Robust Multi alignment Algorithm (RMA). The obtained expression values were analyzed by using the Affymetrix Transcription Analysis Console (TAC) software (Applied Biosystem – Thermo scientific-Italy). Further normalization steps included a per chip normalization to 50th percentile and a per gene normalization to median. Results were filtered for fold change >1.5. Statistical analysis was performed using the ANOVA using as *p*-value cutoff 0.05 and 0.0, respectively. Relative mRNA expression was reported as relative quantitation (RQ) values, calculated as 2^-ΔΔCt^, where Δ*Ct* is calculated as Ct_target gene_ - Ct_housekeeping_ genes (PolR2A mRNA expression). Differences between VPG *vs* CG were considered statistically significant at *p* < 0.05. We used one-way ANOVA calculated with StatView software (version 5.0.1.0; SAS Institute Inc., Cary, NC, United States). Relative protein abundance of ATG5, ATG12, HSP90, HSP70, Bcl-2, and PSMD13 was calculated with respect to GAPDH protein abundance and analyzed with the ANOVA calculated with StatView software (version 5.0.1.0; SAS Institute Inc., Cary, NC, United States).

## Results

### Identification of Differently Expressed Genes (DEGs) in Skeletal Muscle From Veteran Football Players (VPG) Compared to Untrained Subjects (CG)

**Figure 1:**
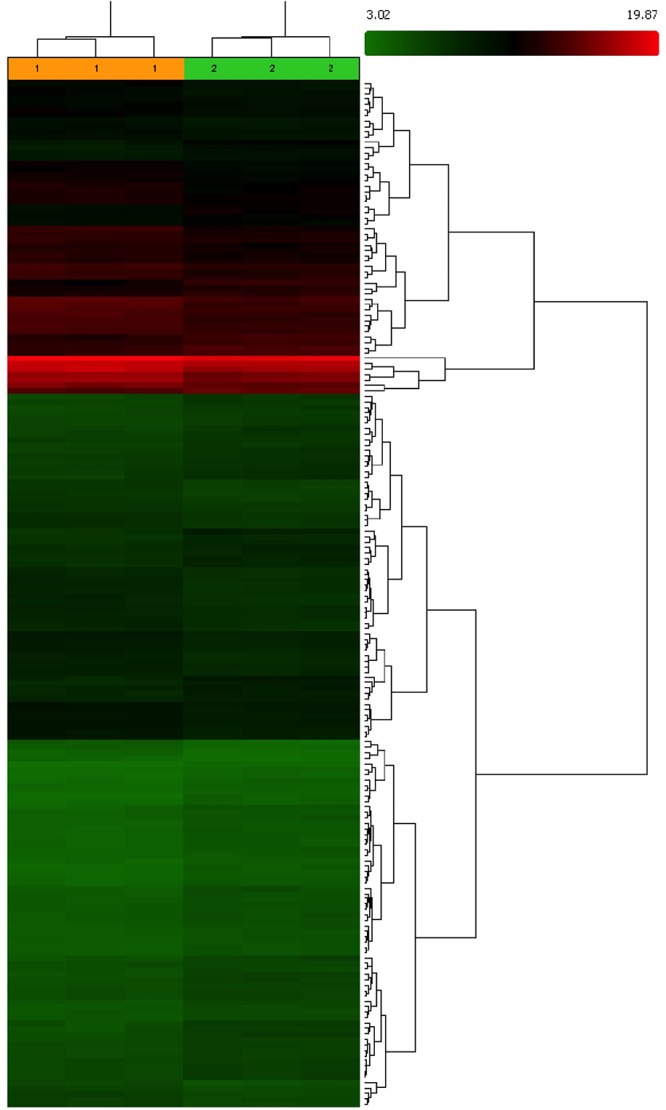
Clustering output of DEGs in skeletal muscle of Veteran Football Players (VPG) compared to untrained (CG) subjects. In Red are up-regulated and in green down-regulated DEGs. A list of transcript identified, fold-change and gene identification is shows in Supplementary Table S1.

We identified the DEGs in skeletal muscle from VPG compared to CG subjects by a GeneChip analysis. After data preprocessing, a total of 430 (*p* < 0.05) and 190 genes (*p* < 0.01), respectively, were identified as differentially expressed between groups. The gene list was further analyzed and a cluster heat map was generated (Figure 1).

### Biological Network Analysis

#### Gene Ontology (GO) Analysis

To understand the function of DEGs, we performed a GO analysis, a commonly used approach for functional studies of large-scale genomic or transcriptomic data. GO terms include biological processes (BP), molecular function (MF), and cellular component (CC). GO analyses were conducted using the on-line software DAVID^5^. The significant enriched GO terms (*p* < 0.05) are listed in Table 2. We identified different GO terms, including 11 biological processes: tricarboxylic acid cycle (GO:0006099), ATP metabolic process (GO:0046034), structural constituent of ribosome (GO:0003735), 4 iron, 4 sulfur cluster binding (GO:0051539), cellular carbohydrate catabolic process (GO:0044275), sarcomere (GO:0030017), nicotinamide metabolic process (GO:0006769), negative regulation of transcription factor activity (GO:0043433), striated muscle contraction (GO:0006941), intracellular organelle lumen (GO:0070013), mitochondrial respiratory chain (GO:0005746).

**Table 2:** Enriched GO terms in biological processes.

GO ID	Gene Name	Count	*p*-value	RG	FDR	Enrichment
GO:0006099	Tricarboxylic acid cycle	5	2.906646E-6	ACO2, CS, IDH2, IDH3B, MDH2	0.000043	6.493506
GO:0046034	ATP metabolic process	6	9.960286E-5	ATP5D, ATP5G2, MYH7, ATP1A2, ATP5I, ATP6V1F	0.001498	7.792207
GO:0003735	Structural constituent of ribosome	6	9.734934E-4	RPL18A, RPLP1, RPL36, MRPL37, RPS9, RPL4	0.012611	7.792207
GO:0051539	4 iron, 4 sulfur cluster binding	3	0.005695	ACO2, NDUFV1, ETFDH	0.071725	3.896103
GO:0044275	Cellular carbohydrate catabolic process	4	0.006330	GPD1L, DLAT, OGDH, MDH2	0.091176	5.194805
GO:0030017	Sarcomere	4	0.009943	TCAP, ANKRD23, MYH7, CACNA1S	0.112608	5.194805
GO:0006769	Nicotinamide metabolic process	3	0.013542	IDH3B, DCXR, MDH2	0.185566	3.896103
GO:0043433	Negative regulation of transcription factor activity	3	0.016946	THRA, PRDX2, COMMD7	0.226872	3.896103
GO:0006941	Striated muscle contraction	3	0.017667	TCAP, MYH7, CACNA1S	0.235357	3.896103
GO:0070013	Intracellular organelle lumen	15	0.025394	ATP5D, ACO2, SRL, CS, RPS9, RPL36, IDH3B, PDLIM1, MYH7, DLAT, OGDH, GOT2, MRPL37, MDH2, VPS25	0.264731	19.480510
GO:0005746	Mitochondrial respiratory chain	3	0.034088	UQCRC1, NDUFB7, NDUFV1	0.339430	3.896103

*GO, Gene Ontology; RG, related genes; FDR, false discovery rate*.

#### Biological Network Identification Affected by Lifelong Football Training in Muscle From VPG by Ingenuity Pathway Analysis (IPA)

To define biological networks affected by lifelong football training, we analyzed the 430 transcripts differentially expressed between the two groups (*p* < 0.05) using the IPA software. IPA output revealed five high-score multidirectional interaction networks. Among them one network was associated with “Small molecule biochemistry, Nucleic acid and amino acid metabolism” (score 79), another one with “Organ Morphology, Nervous System Development and Function” (score 51), and others with “Skeletal and Muscular System Development and Function” (score 25).

**Figure 2:**
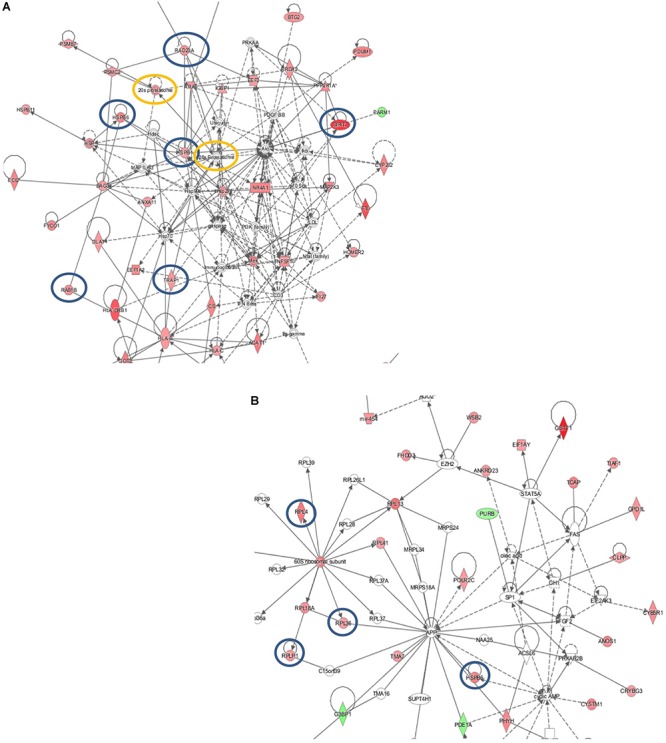
Molecular network generated by the Ingenuity software from the differentially expressed genes in the skeletal muscle from VPG *vs* CG subjects. The main functional pathways given by Ingenuity for this molecular network are: **(A)** “Nucleic Acid, amino acid, and protein Metabolism.” Highlights in this network are the RAD23A, HSPB6, HSB1, Rab1B, TRAP1, SIRT2 genes that are all correlated with increase in auto-lysosomal and proteasome-mediated protein degradation machinery and maintenance of protein quality control. **(B)** “Organ Morphology; Nervous System Development; and Function.” Highlights in this network are ribosomal proteins: RPLP1, RPL4, RPL36, MRLP37 that are involved in cellular growth and proliferation pathways. In red up-regulated and in green down-regulated messenger expression is shown.

**Figure 3:**
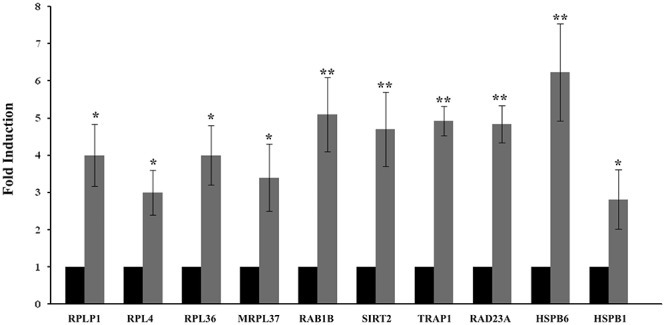
Quantitative expression analysis of representative messengers evidenced by IPA analysis in the skeletal muscle from CG and VPG subjects. Quantitative analysis expression (RT*q*PCR) of RPLP1, RPL4, RPL36, MRPL37, RAB1B, SIRT2, TRAP1, RAD23A, HSPB6, and HSPB1 messenger expression was determined in skeletal muscle biopsies from 15 CG (black bars) to15 VPG subjects (gray bars). An arbitrary value of 1 was assigned to the expression of each messenger in CG. Data represent the means (±SEM) of three different experiments; data were compared using one-way ANOVA and differences were considered significant at ^∗^*p* < 0.05 and ^∗∗^*p* < 0.01 compared to CG.

The results showed that identified DEGs belong to pathways that are involved in different processes correlated to healthy longevity, in particular, Nucleic Acid, amino acid and protein metabolism and Organ Morphology, Nervous System Development and Function (Figure 2A,B). The quantitative analysis by RT-*q*PCR confirmed the over-expression of messengers significantly enriched in the above-mentioned pathways in muscle from VPG. In particular, we found significant up-expression of RAD23A, HSPB6, RAB1B, TRAP1, SIRT2 (*p* < 0.01), HSPB1 (*p* < 0.05) messengers, which are involved in the auto-lysosomal and proteasome-mediated protein degradation machinery and maintenance of protein quality control and similarly, significant up-expression of RPLP1, RPL4, RPL36, MRLP37 (*p* < 0.05) messengers that are involved in cellular growth and proliferation in skeletal muscle from VPG compared to CG (Figure 3).

#### Lifelong-Football Training Affects the Muscle Expression of Rotein Markers Involved in Protein Quality Control Processes

**Figure 4:**
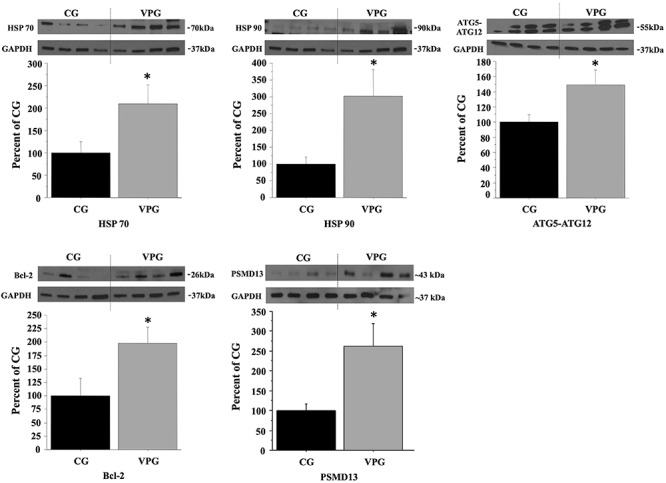
Effects of lifelong football training on the expression of key markers involved in autophagy. Protein expression levels of HSP70, HSP90, and ATG5-ATG12 complex, involved in autophagy process, of anti-apoptotic Bcl-2, and of PSMD13 subunit of the 19S regulator complex were analyzed by Western Blotting in muscle biopsies from 15 controls (CG, black bars) to15 veterans subjects (VPG, gray bars). GAPDH served as loading control. Representative blots are reported for each protein analyzed. Data were expressed as percentage of CG expression. Comparison between groups was determined by ANOVA and represent the means (±SEM) of three different experiments. Differences were considered significant at ^∗^*p* < 0.05 *vs* CG.

To evaluate the effects of lifelong football training on the expression of key proteins involved in proteasome promotion and autophagy, we compared the expression of HSP70, HSP90, ATG5-ATG12 complex, Bcl-2, and PSMD13 proteins in muscle biopsies from VPG *vs* CG subjects. We found significant enhanced expression of all these proteins in muscle from Veterans compared to CG (*p* < 0.05), as reported in Figure 4.

## Discussion

Aging leads to accumulation of damage in functional protein complexes involved in essential cellular processes (Kirkwood, 2002; Wedel et al., 2018). Recently, it has been seen that during aging, an increase in protein damage can be exacerbated by reduced levels of heat shock proteins (HSP) and a diminished autophagy response with consequent impairment of protein quality control (Calderwood et al., 2009).

To our knowledge few studies have investigated the effects of acute and chronic exercise in the elderly on the molecular expression of key markers involved in longevity and on the levels of damaged proteins or organelles in skeletal muscle (Hood et al., 2011; Lira et al., 2013; Cheng et al., 2016; Dickinson et al., 2017; Radak et al., 2018). Autophagy and healthy aging is a new field of research that has not been fully explored until now. This is the first report that investigates the effects of lifelong football training on the autophagy process in particular on the protein biogenesis and anti-apoptotic pathway in skeletal muscle in elderly subjects. We demonstrated that messengers associated with auto-lysosomial and proteasome-mediated pathways were significantly up-regulated in skeletal muscle from VPG compared to CG. In particular we found up-expression of RPL4 and RPL36 messengers, which are regulators of cell-growth and differentiation, promotion of protein quality control and anti-apoptosis pathways in VPG muscle (Blount et al., 2014; Bareh et al., 2016). This is an important finding, as mutations and polymorphisms in RPLs genes are associated with DNA damage and apoptosis in different cells (Wadhwa et al., 2015).

TRAP1 messenger codify for a chaperone protein involved in the reprogramming of cell metabolism following any type of stress. Furthermore, TRAP1 is the only chaperone that directly interacts with mitochondrial respiratory chain components, and also, its expression is correlated to the respiratory capacity of the cells (Lisanti et al., 2014; Matassa et al., 2016). Accordingly, we found the expression of TRAP1 messenger to be up-regulated in VPG compared to CG subjects, which is in line with the improved cardiovascular capacity (VO_2_max) reported for this group (Schmidt et al., 2015). Sirtuins are molecules that connect energy metabolism, oxidative stress, and aging (Karvinen et al., 2016; Zhang et al., 2016). SIRT2 regulates muscle gene expression and differentiation in response to exercise, food-intake, or starvation (Fulco et al., 2003). Over-expression of SIRT2 has been described in endothelial cells associated with the prevention of injuries from high-glucose and improvement of LDL metabolism in mouse models (Zhang W. et al., 2018; Zhang B. et al., 2018). There is growing evidence to indicate, that the expression of Sirtuins 1 and 2 is regulated by exercise in human and animal models, depending on type and duration (Suwa and Sakuma, 2013). However, the observations refer only to short-term bout exercise and no consensus on the topic is established so far. This is the first report investigating the expression of Sirtuins in lifelong trained subjects. We found up-regulation of SIRT2 expression in muscle from VPG subjects associated with improvement of oxidative metabolism, cardiorespiratory capacity and increased expression of key markers correlated to healthy longevity (Krustrup et al., 2010; Mancini et al., 2017).

Protein macromolecules are repeatedly exposed to potential damaging agents during their lifetime, which can cause the loss of molecular function and exhaustion of cell populations. The induction of HSPs is a very important homeostatic adaptation favoring longevity and shielding from damage to intracellular proteins. In addition, HSPs are molecular chaperones which are activated and accumulate in skeletal muscle cells in response to exercise stress-induction (Koh and Escobedo, 2004; Paulsen et al., 2009). In the present study, expression of HSPs was higher in skeletal muscle from elderly trained subjects compared to untrained. This is likely to be a consequence of type, volume and different composition in fiber-type of skeletal muscle, as the presence of oxidative fibers is associated to higher expression of HSPs proteins (Sandri, 2013; Kim et al., 2015). Muscle contraction leads to increased levels of NAD+, AMP, and ROS in the myocytes, which in turn activates downstream signaling effectors as AMPK, p38 kinase, CaMK, Sirtuins (Vainshtein and Hood, 2016). The activation of AMPK by exercise is also requested for the activation of autophagy pathway via Bcl-2 activation (He et al., 2012). The activation of autophagy processes promotes health-metabolic effects and longevity in many tissues including skeletal muscle (Rubinsztein et al., 2011; Madeo et al., 2015; Martinez-Lopez et al., 2015). In association with the activation of MAP-kinase by exercise, PGC1-α up-regulation and suppression of mTOR expression are other key signals associated with the activation of autophagy processes (Vainshtein and Hood, 2016). We previously demonstrated that lifelong football training induces the expression of AMPK, ERK1,2 and p38kinase proteins in muscle from VPG compared to elderly untrained subjects (Mancini et al., 2017). Here, we report the up-regulation of HSPB6, HSPB1, HSP70, and HSP90 subunits, usually induced when cells are exposed to protein damaging agents, in muscle from VPG subjects. HSP70 and HSP90 mediate the check-up of the protein quality control and help cells to avoid damage-dependent apoptosis; in particular, HSP90 subunit is the key component in the assembly of the lysosomal-membrane protein type 2A complex (LAMP-2A), involved in the chaperone-mediated autophagy pathway (Salminen and Kaarniranta, 2009). Also, we found increased in the expression of ATG5-ATG12 complex and Bcl-2 protein, both positively related to authophagy pathway. In particular, the ATG5-ATG12 complex is essential for autophagosome assembly (Cuervo, 2008; Walczak and Martens, 2013) and the anti-apoptotic Bcl-2 protein plays important roles in the crosstalk between autophagy and apoptosis, being able to cooperate with ATG5 to committee the muscle cells toward autophagy if up-regulated, or toward apoptosis if down-regulated (Zhou et al., 2011). Interestingly, we also found that the expression of HSPs, Bcl-2, and ATG5-ATG12 complexes parallels the up-regulation of AMPK, PGC1-α in muscle from VPG, as previously demonstrated (Mancini et al., 2017).

Finally, we found up-regulation of RAD23A messenger expression in muscle from VPG subjects; RAD proteins are involved in the Nucleic Excision Repair process (Shuck et al., 2008), in Ubiquitin-dependent protein degradation pathway (Yokoi and Hanaoka, 2017) and in the interaction with proteasome complex finalized to delivery of ubiquitinated proteins (Liang et al., 2014). We also found the overexpression of the PSMD13 protein, named also Rpn9, a non-ATPase subunit of the 19S regulator complex, which is involved in the assembly and/or stability of the 26S proteasome (Takeuchi et al., 1999).

All these observations strongly support the hypothesis that lifelong football training enhances autophagy and in general a more efficient protein quality control processes avoiding damaged protein accumulation in skeletal muscle of elderly subjects, in turn promoting longevity.

## Conclusion

We demonstrated that lifelong football training is able to induce transcriptional activation of key markers involved in pathways of protein quality control, in particular autophagy, and associated to improvement of intermediate metabolism and cardiovascular capacity in elderly VPG compared to untrained subjects. There is growing evidence to indicate that the expression of HSPs and autophagy process are activated by acute or chronic exercise. Increased levels of molecular chaperones and autophagy process play an important role in preventing protein damage during aging and are associated with longevity, but the molecular mechanisms leading to these effects have not been completely elucidated so far. Moreover, no consensus has been reached on exercise-type, intensity, or volume regarding the activation of this process, the effects on improvement of intermediate metabolism and longevity promotion. In this scenario, this was the first attempt to evaluate the effects mediated by lifelong football training on the autophagy and healthy aging pathway. Future studies should aim to include larger sample sizes and test the effects of post-transcriptional and epigenetic factors that contribute to the autophagy process activation mediated by football training.

## Author Contributions

AmM and PB conceived the manuscript, and made contributions on acquisition, analysis, and interpretation of data. MR, MH, TA, JS and PK enrolled the subjects and collected the muscle samples. SO performed bioinformatic analysis. DV, EI, and AlM carried out the experiments and performed statistical analysis of data.

## Conflict of Interest Statement

The authors declare that the research was conducted in the absence of any commercial or financial relationships that could be construed as a potential conflict of interest.

## Footnotes

1 https://www.gov.uk/government/uploads/system/uploads/attachment_data/file/213741/dh_128146.pdf

2 https://www.ncbi.nlm.nih.gov/geo/query/acc.cgi?acc=GSE125830

3 https://david.ncifcrf.gov/

4 http://www.ingenuity.com

5 https://david.ncifcrf.gov/
