# Supplementary material for: Lifelong Football Training: Effects on Autophagy and Healthy Longevity Promotion
Source: Front Physiol. 2019 Feb 19;10:132. doi: 10.3389/fphys.2019.00132 (PMC6390296; doi:10.3389/fphys.2019.00132)
Supplement: Supplementary file 1 [file Data_Sheet_1.PDF]

Table1S: List of DEGs in skeletal muscle of VPG *vs* CG subjects, fold-change and gene symbol

Transcript Cluster ID	Fold Change	*p-value*	Gene Symbol
TC0X001621.hg.1	-1.51	0.000050	GS1-433O24.1
TC08001642.hg.1	-1.78	0.000065	RNU6-1255P
TC19000792.hg.1	1.58	0.000135	PPP2R1A
TC16000825.hg.1	1.98	0.000145	SRL
TC19002477.hg.1	3.24	0.000197	SIRT2
TC20000146.hg.1	1.52	0.000279	LOC100652902
TC20001032.hg.1	1.87	0.000447	EEF1A2
TC01001837.hg.1	-3.85	0.000476	RNU6-1248P
TC15001841.hg.1	2.19	0.000488	IDH2
TC03000157.hg.1	1.68	0.000503	GPD1L
TC12000494.hg.1	-1.64	0.000528	RNU1-69P
TC09001307.hg.1	-2.54	0.000569	
TC14000569.hg.1	-1.67	0.000600	RNU6-366P
TC02000029.hg.1	-1.87	0.000606	RNU6-649P
TC16000490.hg.1	1.62	0.000614	COQ9
TC18000495.hg.1	-1.74	0.000615	RNU6-1131P
TC02000317.hg.1	-1.70	0.000668	RPL23AP32
TC16001114.hg.1	-2.05	0.000721	
TC19000344.hg.1	1.71	0.000741	RPL18A
TC05002903.hg.1	-1.56	0.000760	
TC19000233.hg.1	1.73	0.000870	RAD23A
TC19000104.hg.1	1.52	0.000871	RPL36
TC14001930.hg.1	1.55	0.000876	MYH7
TC10001549.hg.1	1.98	0.000907	PDLIM1
TC01005016.hg.1	-1.64	0.000940	
TC11001615.hg.1	-1.52	0.000988	
TC05000003.hg.1	1.69	0.001029	LRRC14B
TC19002217.hg.1	1.86	0.001044	PPP2R1A
TC07001774.hg.1	-1.55	0.001071	
TC19000694.hg.1	-1.57	0.001138	SNAR-C3
TC17000535.hg.1	1.63	0.001175	VPS25
TC15000348.hg.1	-3.27	0.001209	RNU6-1332P
TC0X001760.hg.1	-1.94	0.001218	PABPC1L2A
TC02001088.hg.1	1.52	0.001230	RNA5SP113
TC19001240.hg.1	2.06	0.001230	NDUFB7
TC06001651.hg.1	-2.27	0.001231	
TC01001870.hg.1	-1.65	0.001236	RNA5SP77
TC04000321.hg.1	-2.00	0.001261	
TC09000205.hg.1	-1.53	0.001317	RNU4-53P
TC22001502.hg.1	2.07	0.001343	CPT1B
TC10001912.hg.1	-2.65	0.001344	RP11-214D15.2
TC05000142.hg.1	-2.00	0.001362	RNU6-378P
TC04000806.hg.1	1.58	0.001394	ETFDH
TC09002111.hg.1	1.77	0.001415	
TC01002108.hg.1	1.64	0.001418	TMEM52
TC15000097.hg.1	-2.02	0.001703	SNORD115-27
TC14001531.hg.1	1.66	0.001764	ANKRD9
TC17000060.hg.1	1.52	0.001768	KIF1C
TC22000337.hg.1	1.79	0.001835	ACO2

Transcript Cluster ID	Fold Change	*p-value*	Gene Symbol
TC16000286.hg.1	-1.74	0.001866	RNA5SP405
TC11001882.hg.1	-1.86	0.001953	RNU6-118P
TC02001710.hg.1	-1.73	0.002034	
TC20000766.hg.1	1.53	0.002100	COMMD7
TC16000224.hg.1	-1.81	0.002203	RNU6-944P
TC01002537.hg.1	-1.81	0.002209	RNA5SP44
TC06000803.hg.1	-1.52	0.002215	RP11-63K6.7
TC10000335.hg.1	-1.89	0.002240	
TC10001283.hg.1	-1.89	0.002240	
TC04001198.hg.1	-2.66	0.002267	RNU6-310P
TC15000639.hg.1	1.59	0.002281	RPLP1
TC05001064.hg.1	-1.65	0.002353	RNU1-17P
TC17002875.hg.1	2.00	0.002383	TCAP
TC0X002171.hg.1	-1.61	0.002448	
TC12001813.hg.1	2.19	0.002454	
TC01001269.hg.1	1.56	0.002458	HAX1
TC19002231.hg.1	2.63	0.002526	RPS9
TC0X001416.hg.1	1.63	0.002544	ARHGEF6
TC03000306.hg.1	1.70	0.002557	SLC38A3
TC0M000008.hg.1	-1.86	0.002557	
TC15001265.hg.1	-2.29	0.002598	RNU6-188P
TC0X000237.hg.1	1.57	0.002606	ARAF
TC14001252.hg.1	-1.63	0.002653	RNU6-921P
TC05001026.hg.1	-1.71	0.002756	
TC10000231.hg.1	-1.56	0.002793	
TC19000450.hg.1	-2.06	0.002858	
TC05002527.hg.1	1.58	0.002904	CAST
TC19000032.hg.1	2.34	0.002946	ATP5D
TC22000400.hg.1	-1.57	0.002984	
TC11002881.hg.1	-1.61	0.003003	
TC18000218.hg.1	-2.35	0.003066	RNU6-116P
TC22001420.hg.1	2.17	0.003119	CHKB-CPT1B
TC03000403.hg.1	-3.29	0.003178	MIR548A2
TC02001091.hg.1	-2.28	0.003185	RNU6-1122P
TC11000696.hg.1	1.85	0.003313	NDUFV1
TC11000665.hg.1	-1.56	0.003362	RNU1-84P
TC06003146.hg.1	-2.70	0.003401	
TC01000097.hg.1	-1.63	0.003461	RNU1-8P
TC22000669.hg.1	-1.51	0.003496	RNU6-338P
TC12001263.hg.1	-1.59	0.003558	RNA5SP353
TC06001931.hg.1	-2.28	0.003679	
TC17000393.hg.1	-1.83	0.003696	RNU6-840P
TC03001589.hg.1	-1.82	0.003763	
TC10000601.hg.1	-1.59	0.003794	RNU6-325P
TC01001142.hg.1	-1.63	0.003832	RNVU1-14
TC0X000461.hg.1	-2.18	0.003852	
TC16001930.hg.1	1.57	0.003942	FAM96B
TC15000907.hg.1	2.33	0.003952	
TC05000237.hg.1	-1.76	0.004028	RNA5SP183

Transcript Cluster ID	Fold Change	*p-value*	Gene Symbol
TC11000626.hg.1	-1.84	0.004072	
TC02004291.hg.1	-1.62	0.004282	
TC11000863.hg.1	-1.62	0.004349	RNU6-1292P
TC07000276.hg.1	1.86	0.004355	OGDH
TC15000346.hg.1	-2.02	0.004356	RNU1-119P
TC12002893.hg.1	2.15	0.004368	ATP5G2
TC17002195.hg.1	1.77	0.004431	THRA
TC01000663.hg.1	1.74	0.004541	MRPL37
TC01005482.hg.1	1.52	0.004554	
TC19000459.hg.1	1.51	0.004615	FXYD1
TC18000506.hg.1	2.40	0.004715	ACAA2
TC11002077.hg.1	-2.14	0.004726	RNA5SP343
TC08001316.hg.1	1.56	0.004773	EYA1
TC0X001119.hg.1	-1.62	0.004850	RNU1-56P
TC11000657.hg.1	1.76	0.004874	RAB1B
TC12003213.hg.1	1.92	0.004875	CS
TC01001978.hg.1	-1.51	0.004886	RP11-561I11.2
TC07000154.hg.1	-1.77	0.004887	RNU6-1103P
TC08000469.hg.1	-1.58	0.004962	
TC02002953.hg.1	1.51	0.004972	THAP4
TC03000631.hg.1	1.62	0.005156	FAM162A
TC11002866.hg.1	1.57	0.005190	DLAT
TC03001384.hg.1	1.74	0.005199	UQCRC1
TC17001583.hg.1	-1.56	0.005244	
TC11003183.hg.1	-1.85	0.005272	
TC12000289.hg.1	-2.49	0.005352	RNU6-318P
TC08002112.hg.1	1.55	0.005375	
TC09001951.hg.1	1.55	0.005471	
TC05001337.hg.1	-2.10	0.005583	RNU6-480P
TC08001621.hg.1	-1.99	0.005650	RP11-622O11.4
TC02001432.hg.1	-1.87	0.005667	
TC02000343.hg.1	-1.78	0.005710	RNU1-32P
TC01003684.hg.1	1.91	0.005757	CACNA1S
TC07000746.hg.1	1.59	0.005973	LMOD2
TC11003499.hg.1	1.91	0.006026	COX8A
TC15001644.hg.1	1.64	0.006183	UBL7
TC22000766.hg.1	-2.39	0.006334	
TC03000540.hg.1	-1.94	0.006358	
TC20000709.hg.1	-1.69	0.006437	RNA5SP479
TC03001353.hg.1	1.84	0.006493	FYCO1
TC02000596.hg.1	-2.28	0.006668	
TC20000036.hg.1	-2.38	0.006693	RNU6-1019P
TC19001210.hg.1	1.67	0.006794	PRDX2
TC20001384.hg.1	2.11	0.006824	IDH3B
TC15002586.hg.1	2.12	0.007094	RPL4
TC15001869.hg.1	-1.57	0.007108	
TC10000591.hg.1	1.67	0.007199	GHITM
TC21000341.hg.1	-1.93	0.007226	RNU6-123P
TC09002184.hg.1	1.93	0.007362	HSDL2
TC19001453.hg.1	1.97	0.007372	HSPB6

Transcript Cluster ID	Fold Change	*p-value*	Gene Symbol
TC02001380.hg.1	-1.99	0.007571	RNU6-268P
TC07000786.hg.1	1.56	0.007594	ATP6V1F
TC08000621.hg.1	-1.69	0.007600	RNU6-1092P
TC16000822.hg.1	1.63	0.007602	TRAP1
TC17001315.hg.1	1.59	0.007647	TIAF1
TC06002724.hg.1	1.52	0.007698	RNF5P1
TC17001035.hg.1	1.84	0.007724	SLC25A11
TC04000822.hg.1	-1.61	0.007780	RNU6-668P
TC05000854.hg.1	-1.88	0.007843	MIR1303
TC02002849.hg.1	-1.66	0.007915	RNU6-964P
TC01001361.hg.1	1.55	0.007923	ATP1A2
TC18000319.hg.1	-1.55	0.007930	RNU6-903P
TC04000951.hg.1	1.63	0.007931	ATP5I
TC01002830.hg.1	-2.05	0.008025	
TC11000227.hg.1	-1.67	0.008029	
TC14000279.hg.1	-1.62	0.008107	RNU6-539P
TC11001035.hg.1	-1.69	0.008258	
TC07000479.hg.1	1.95	0.008274	MDH2
TC05001569.hg.1	-1.51	0.008382	RNU6-606P
TC11002832.hg.1	-1.52	0.008419	
TC19000531.hg.1	1.81	0.008467	EIF3K
TC01000704.hg.1	-2.91	0.008483	RNU6-414P
TC07002919.hg.1	1.83	0.008592	POLR2J4
TC0X000440.hg.1	-2.67	0.008715	
TC05002656.hg.1	1.96	0.008752	CYSTM1
TC6_cox_hap2000236.h	1.51	0.008831	HLA-C
TC09001446.hg.1	-1.62	0.008839	RNU6-492P
TC07001329.hg.1	1.69	0.008908	POLR2J4
TC09000356.hg.1	-1.55	0.008933	RN7SKP59
TC01003213.hg.1	-2.12	0.008949	RNU6-1309P
TC08000639.hg.1	-2.01	0.008971	KB-1507C5.3
TC19002531.hg.1	1.51	0.009163	NAPA
TC04002406.hg.1	2.15	0.009246	ATP5I
TC08002539.hg.1	1.90	0.009324	ST3GAL1
TC02002993.hg.1	-1.89	0.009417	AC019118.4
TC02002449.hg.1	-1.99	0.009535	
TC17001970.hg.1	1.99	0.009544	DCXR
TC09002030.hg.1	1.59	0.009568	
TC08000647.hg.1	-1.55	0.009615	RNU6-1011P
TC02005037.hg.1	1.62	0.009666	ANKRD23
TC16001158.hg.1	2.00	0.009705	GOT2
TC08000110.hg.1	-1.81	0.009741	RNU6-842P
